# Urinary N telopeptide levels in predicting the anti-nociceptive responses of zoledronic acid and paclitaxel in a rat model of bone metastases

**DOI:** 10.3892/mmr.2015.3948

**Published:** 2015-06-17

**Authors:** QI GUI, CHENGCHENG XU, DAPENG LI, LIANG ZHUANG, SHU XIA, SHIYING YU

**Affiliations:** 1 Departments of Oncology, The First Affiliated Hospital of Soochow University, Suzhou, Jiangsu 215006, P.R. China; 2 Departments of Thoracic Surgery, The First Affiliated Hospital of Soochow University, Suzhou, Jiangsu 215006, P.R. China; 3 Department of Oncology, Tongji Hospital, Huazhong University of Science and Technology, Wuhan, Hubei 430030, P.R. China

**Keywords:** Type I collagen-crosslinked N telopeptide, paclitaxel, zoledronic acid, anti-nociceptive effects, bone metastases

## Abstract

The present study investigated the hypothesis that urinary levels of N telopeptide (NTx) can be used to predict the anti-nociceptive responses of zoledronic acid and paclitaxel on bone metastases in a rat model. Rats were implanted with intra-femur Walker 256 carcinoma cells or control solution, and were treated with either normal saline, zoledronic acid or paclitaxel on the 10th day following surgery. Mechanical allodynia was recorded and the urine collagen-crosslinked NTx values were measured prior to, and 7, 14 and 21 days following the injections. Bone sections and osteoclasts were stained on the 14th day (4 days post-injection). Furthermore, the mRNA and protein expression levels of c-fos in the spinal cord and acid-sensing ion channel 3 (ASIC3) in the dorsal root ganglion (DRG) were analyzed. The mechanical allodynia of rats was attenuated from day 14 in the zoledronic acid group and from day 21 in the paclitaxel group. A positive correlation was observed between the anti-nociceptive responses of zoledronic acid and paclitaxel, and the urinary levels of NTx (r=0.619; P<0.001). The mRNA levels of c-fos in the spinal cord and ASIC3 in the DRG in the zoledronic acid group were reduced 14 and 21 days after inoculation, and this reduction was observed in the paclitaxel group 21 days after inoculation. Low dose paclitaxel was observed to have a weaker anti-nociceptive effect on bone cancer pain, with a later-onset, compared with zoledronic acid. The results suggested that urinary levels of NTx may predict the anti-nociceptive responses of zoledronic acid and paclitaxel in a rat model of bone metastases.

## Introduction

Bone is the most common organ for advanced-stage solid tumor metastasis, particularly those arising from the breast, prostate and multiple myeloma (1). Bone metastases disrupts skeletal metabolism and results in potentially debilitating or life-limiting skeletal-associated events, including intractable, chronic bone pain (2). Several approaches have been suggested to treat advanced-stage solid tumors and skeletal complications, including radiation therapy, chemotherapy, bisphosphonates and analgesia (3). Using these strategies, previous studies have demonstrated that zoledronic acid, a third-generation bisphosphonate, improves the control of bone pain in patients with cancer (4,5). Paclitaxel, a chemotherapeutic drug, reduces the growth of osteolytic lesions and exhibits anti-tumor and anti-resorptive effects in bone metastases in rat models (6). These effects may also reduce bone pain in patients with cancer.

At present, the anti-nociceptive effect of paclitaxel of bone metastases remains to be fully elucidated. In addition, due to the combined use of opioid analgesics or non-steroidal anti-inflammatory drugs, the anti-nociceptive effect of zoledronic acid and paclitaxel for metastatic bone pain is often masked and, therefore, remains unclear. Therefore, it is of interest to investigate the respective analgesic effects of zoledronic acid and paclitaxel in bone metastases.

Previous studies have demonstrated that the Type I collagen-crosslinked N telopeptide (NTx) is a bone absorption marker for the occurrence and progression of bone metastases, which can be used to predict the prognosis of bone disease (7,8). Therefore, the present study aimed to investigate the correlation between urinary levels of NTx and the anti-nociceptive responses of zoledronic acid and paclitaxel in bone metastases using a rat model, and to examine the potential underlying mechanisms.

## Materials and methods

### Preparation of cells

Walker 256 rat mammary gland carcinoma cells were provided by the Laboratory of the Department of Anesthesiology in Tongji Hospital (Huazhong University of Science and Technology, Wuhan, China). The cells (1×10^6^/ml) were cultured in RPMI 1640 medium (Gibco Life Technologies, Carlsbad, CA, USA), supplemented with 10% fetal bovine serum and 2% penicillin/streptavidin in a humidified atmosphere of 5% CO_2_. The cells were collected by centrifugation for 3 min at 240 × g, rinsed with calcium-and magnesium-free Hank's solution, counted with a hemocytometer (DHC-N01; Incyto, Cheonan, Korea) and re-centrifuged under the same conditions. The cells were diluted to a final concentration for femoral inoculation of 10^5^ cells in 10 *µ*l Hank's solution, and were maintained on ice prior to surgery.

### Animals and induction of bone metastases

Female Wistar rats 7 weeks-old, weighing 220-250 g (n=8/group; Experimental Animal Research Center, Wuhan, China; certificate no. SCXK E 2008-0005) were housed in a specific pathogen-free room (22±0.5°C; 12-h light/dark cycle; food and water *ad libitum*). All experiments were performed in accordance with the ethical guidelines of the National Institutes of Health (9), and the present study was approved by the ethics committee of Soochow University (Suzhou, China).

The surgical procedure for the induction of bone metastases was the same as that reported previously by Gui *et al*. Briefly, the rats were fully anesthetized with 6% chloral hydrate (Sinopharm Chemical Reagent Co., Ltd., Shanghai, China) and laid in the prone position. Subsequent to shaving and disinfection of the left leg, a 1 cm-long incision was made over the third trochanter and rectus femoris muscle along the medical edge, to expose the shaft of the femur. A needle was then inserted vertically into the shaft of femur to reach the intramedullary canal of the femur. The needle was then replaced with a micro-injection syringe containing 10 *µ*l Walker 256 cells (10^5^ cells) or Hank's solution (sham group). Following slow injection and a 2 min delay to allow cell dispersion within the bone marrow, the syringe was removed and the drill hole was sealed using bone wax (Friends of Shanghai Medical Devices Co., Ltd., Shanghai, China). The site was thoroughly washed with sterile de-ionized water. The muscle and skin were finally stitched and disinfected.

### Drug administration

The rats were randomly divided into six groups (8 rats/group). The first two groups were the healthy control group (naive) and surgical procedure control group (sham). In the remaining four groups, the animals were allowed to develop bone metastasis for 10 days following the implantation of Walker 256 cells. The groups were defined, as follows: Cancer control (no-therapy); cancer with NS (intraperitoneal injection of normal saline on day 10); cancer with ZOL, involving subcutaneous administration of 0.1 mg/kg zoledronic acid (Zometa^®^; Novartis Pharma Schweiz AG, Rotkreuz, Switzerland), on day 10; and cancer with TA, involving intra-peritoneal injection of 5 ml, 30 mg and 2 mg/kg paclitaxel (Taxol^®^; Bristol-Myers Squibb S.R.L., Rome, Italy), on day 10.

### Mechanical allodynia

Mechanical allodynia was assessed using a dynamic plantar aesthesiometer (21025; Ugo Basile S.R.L., Comerio, Italy) as described previously (10), which is an automated von Frey-type system. Briefly, the rats were placed individually into elevated wire mesh bottomed enclosures, and a straight metal filament was pushed against the hind paw with increasing force. When the animal withdrew its hind paw or the preset cut-off was reached (50 g), the force was automatically registered in grams and the filament was automatically removed.

### Measurements of urinary Type I collagen-crosslinked NTx

Bone resorption was assessed by measuring the levels of urinary NTx, associated with creatinine (nmol/mmol/creatinine). The rats were placed in metabolic cages each week to collect 24 h urine. Freshly collected urine was centrifuged at 670 × g for 5 min. The supernatant was frozen at −80°C until required for analysis of the NTx levels using a commercially available enzyme-linked immunosorbent assay (ELISA) kit (Rat cross linked N-telopeptide of type I collegan, NTX ELISA kit; cat. no. CSB-E09243r; Cusabio Biotech Co., Ltd., Wuhan, China), according to the manufacturer's instructions. Each sample was assayed in duplicate.

### Histological analysis

The mice were sacrificed by CO_2_ inhalation, and the femoral bones were dissected and the specimens were fixed in 4% paraformaldehyde for 2 days and decalcified in 10% EDTA for 3 weeks. Subsequent to embedding in paraffin, the sections were cut into 4 *µ*m-sections and stained with Harris' hematoxylin and eosin (H&E; Beyotime Institute of Biotechnology, Haimen, China). The femoral sections were then subjected to staining for tartrate-resistant acid phosphatase (TRAP; Nanjing Jiancheng Bioengineering Institute, Nanjing, China), according to the manufacturer's instructions. TRAP-positive cells were observed under an inverted microscope (IX53; Olympus Corporation, Tokyo, Japan) and five randomly selected fields were analyzed.

### Reverse transcription-quantitative polymerase chain reaction (RT-qPCR) analysis

The total RNA was extracted from the L4–6 spinal cord and dorsal root ganglion (DRG) using TRIzol reagent (Takara Biotechnology Co., Ltd., Dalian, China), according to the manufacturer's instructions. Total RNA (2 *µ*g) was used for RT to synthesize cDNA using SuperScriptase III (Thermo Fisher Scientific, Burlington, ON, Canada) with random primers. To detect the mRNA expression levels of c-fos, TRPV1 and ASIC3, a SYBR Green method (SYBR^®^ Green PCR Master Mix; Thermo Fisher Scientific) was used with the following primers: C-fos, forward TGC CAA TCT ACT GAA AGA GA and reverse TCCAGGGAGGTCACAGAC; TRPV1, forward GGAAGACAGACAGCCTGA and reverse ATCTGCTCCATTCTCCAC; and ASIC3, forward AATACCGCATCTTTGGAT and reverse CACATAGCGAGACTCACAG. RT-qPCR analysis of gene expression was performed using an ABI 7900 Sequence Detector (Applied Biosystems Life Technologies, Foster City, CA, USA). The thermal cycling conditions included 40 cycles of 95°C for 15 sec and 55°C for 1 min. The relative value of target mRNA expression was normalized to the expression of GAPDH, calculated using the 2^ΔΔCT^ method and compared with the control group.

### Western blot analysis

L4–6 spinal cord tissues were collected from each group of animals. The total protein of the ipsilateral spinal cords was extracted using radioimmunoprecipitation assay protein lysis buffer (Beyotime Institute of Biotechnology). Protein concentration was determined using a bicinchoninic acid assay kit (Beyotime Institute of Biotechnology). Equal quantities of protein (200 *µ*g) were fractionated using SDS-PAGE (10%; Beyotime Institute of Biotechnology), transferred onto polyvinylidene difluoride membranes (Merck Millipore, Darmstadt, Germany) and then blocked with 10% non-fat dry milk for 3 h at room temperature. Primary antibodies for c-fos (cat. no. sc-52), TRPV1 (cat. no. sc-28759; polyclonal rabbit anti-rat; 1:1,000; Santa Cruz Biotechnology, Inc., Dallas, TX, USA) and GAPDH (monoclonal rabbit anti-rat; 1:10,000; Epitomics, Burlingame, CA, USA) were incubated overnight at 4°C, followed by probing for 2 h at room temperature with an anti-rabbit horseradish peroxidase-conjugated secondary antibody (1:5,000; Santa Cruz Biotechnology, Inc.). Finally, the immunoreactive bands were visualized in enhanced chemiluminescence solution (Pierce Biotechnology, Inc., Rockford, IL, USA) and exposed onto X-ray films.

### Statistical analysis

SPSS software, version 12.0 (SPSS, Inc., Chicago, IL, USA) was used to perform statistical analysis. One-way analysis of variance, followed by Bonferroni's post-hoc test, was used for statistical comparison among the various groups. P<0.05 was considered to indicate a statistically significant difference (Eastman Kodak, Rochester, NY, USA).

## Results

### Zoledronic acid and paclitaxel attenuate mechanical allodynia

**Figure 1:**
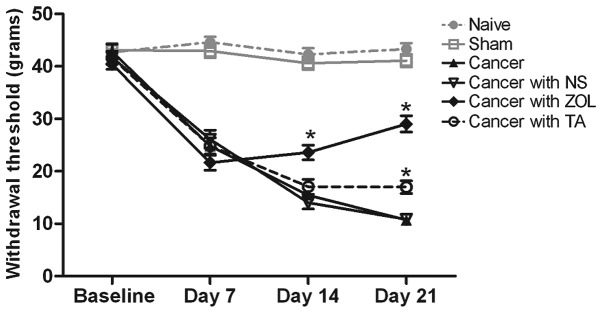
Effects of zoledronic acid and paclitaxel on mechanical allodynia in the bone metastases model. ^*^P<0.05, compared with the sham group. NS, normal saline; ZOL, zoledronic acid; TA, paclitaxel.

Significant reductions in the withdrawal thresholds of the dynamic von Frey test (indicating allodynia) were observed by day 7 following intrafemur inoculation of the Walker 256 rat mammary gland carcinoma cells (P<0.05; Fig. 1). Compared with the cancer group and cancer with NS group following the administration of zoledronic acid on day 10 post-implantation, the mechanical allodynia of the rats were significantly attenuated from day 14 onwards (P<0.05). In the cancer with TA group, the rats exhibited a reversal of the altered withdrawal response on day 21, whereas a significant reduction in allodynia was observed in the cancer with ZOL group (P<0.05).

### Correlation between the urinary levels of NTx and the anti-nociceptive responses of zoledronic acid and paclitaxel

**Figure 2:**
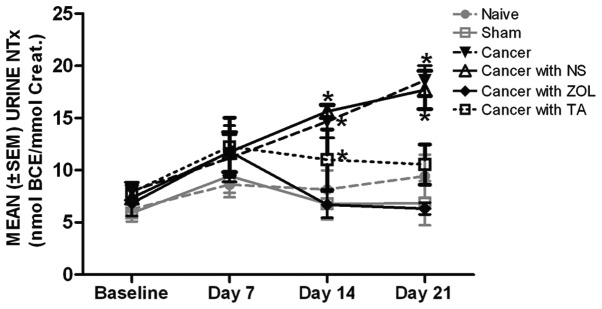
Alterations in urinary levels of NTx following administration of zoledronic acid and paclitaxel. ^*^P<0.05, compared with the sham group. NTx, N telopeptide; NS, normal saline; ZOL, zoledronic acid; TA, paclitaxel; SEM, standard error of the mean; BCE, bone collagen equivalents; Creat, creatinine.

Analyses of the urinary levels of NTx from each group of rats is shown in Fig. 2. At baseline and day 7, no significant difference were observed in the urinary levels of NTx among each group (P=0.824). At day 14 post-inoculation of cancer cells (day 4 post-administration), the urinary levels of NTx were significantly increased in the cancer group, cancer with NS group and cancer with TA group, compared with the naive and sham groups (P<0.05), whereas no significant differences were observed between the urinary levels of NTx in the cancer with ZOL group and the naive or sham groups. In the cancer with TA group, the urinary level of NTx was significantly reduced, compared with the cancer group and cancer with NS group on day 21 post-implantation (day 11 post-administration). Collectively, these results suggested that the reduced effect of zoledronic acid on urinary levels of NTx occurred earlier than that of paclitaxel. Furthermore, a positive correlation was observed between the anti-nociceptive responses of zoledronic acid and paclitaxel and the urinary levels of NTx, which was statistically significant (correlation coefficient=0.619; P<0.001).

### Histological alterations in tumor growth following zoledronic acid and paclitaxel treatment

**Figure 3:**
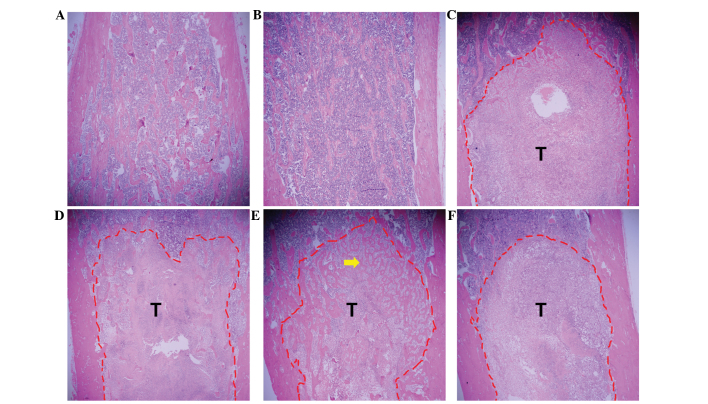
Histological evaluation of tumor growth 14 days post-tumor cell inoculation. Histological sections in (A) naive, (B) sham, (C) cancer, (D) cancer with NS, (E) cancer with ZOL and (F) cancer with TA groups. T marks the tumor, the red dotted line outlines the extent of the tumor and the yellow arrow indicates trabecular bone. NS, normal saline; ZOL, zoledronic acid; TA, paclitaxel.

The bones in the naive and sham control groups exhibited no infiltration of the bone marrow space by malignant tumors. On day 14 post-tumor cell inoculation (day 4 post-administration of saline, zoledronic acid or paclitaxel), all the rats, which were injected with Walker 256 cells developed tumors, which were visible in the cancer, cancer with NS, cancer with ZOL and cancer with TA groups (Fig. 3). The extent of bone marrow replacement by the tumor in each cancer group was similar (red dotted line). However, in the cancer with ZOL group, more residual trabecular bones within the tumor (yellow arrow) were observed, compared with the cancer, cancer with NS and cancer with TA groups.

### Zoledronic acid treatment significantly reduces osteoclast numbers

**Figure 4:**
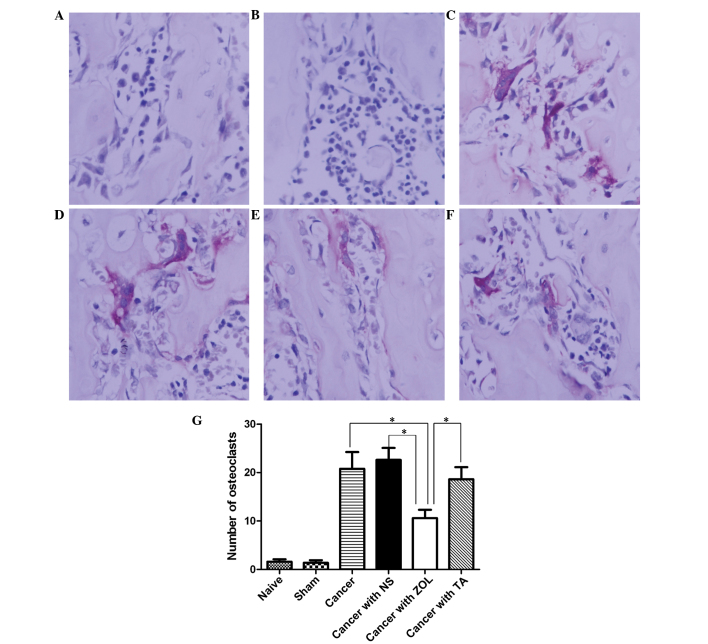
Effects of zoledronic acid and paclitaxel treatments on osteoclast number. Representative TRAP staining profiles of (A) naive, (B) sham, (C) cancer, (D) cancer with NS, (E) cancer with ZOL and (F) cancer with TA groups (magnification, ×400). (G) Number of osteoclasts (multinucleate TRAP-positive cells) were counted under a microscope in five randomly-selected fields (magnification of ×100). ^*^P<0.05. TRAP, tartrate resistant acid phosphatase; NS, normal saline; ZOL, zoledronic acid; TA, paclitaxel.

The femur sections on day 14 post-tumor cell inoculation (day 4 post-administration of saline, zoledronic acid or paclitaxel), were stained for TRAP to identify osteoclasts (Fig. 4). Few TRAP-positive cells were observed in the naïve and sham groups, compared with which, the number of osteoclasts in each cancer group was increased significantly (P<0.05). ZOL treatment resulted in significantly fewer osteoclasts, compared with the cancer group, cancer with NS group and cancer with TA group (P<0.05).

### Zoledronic acid and paclitaxel significantly reduce the relative mRNA expression levels of c-fos in the spinal cord and ASIC3 in the DRG

**Figure 5:**
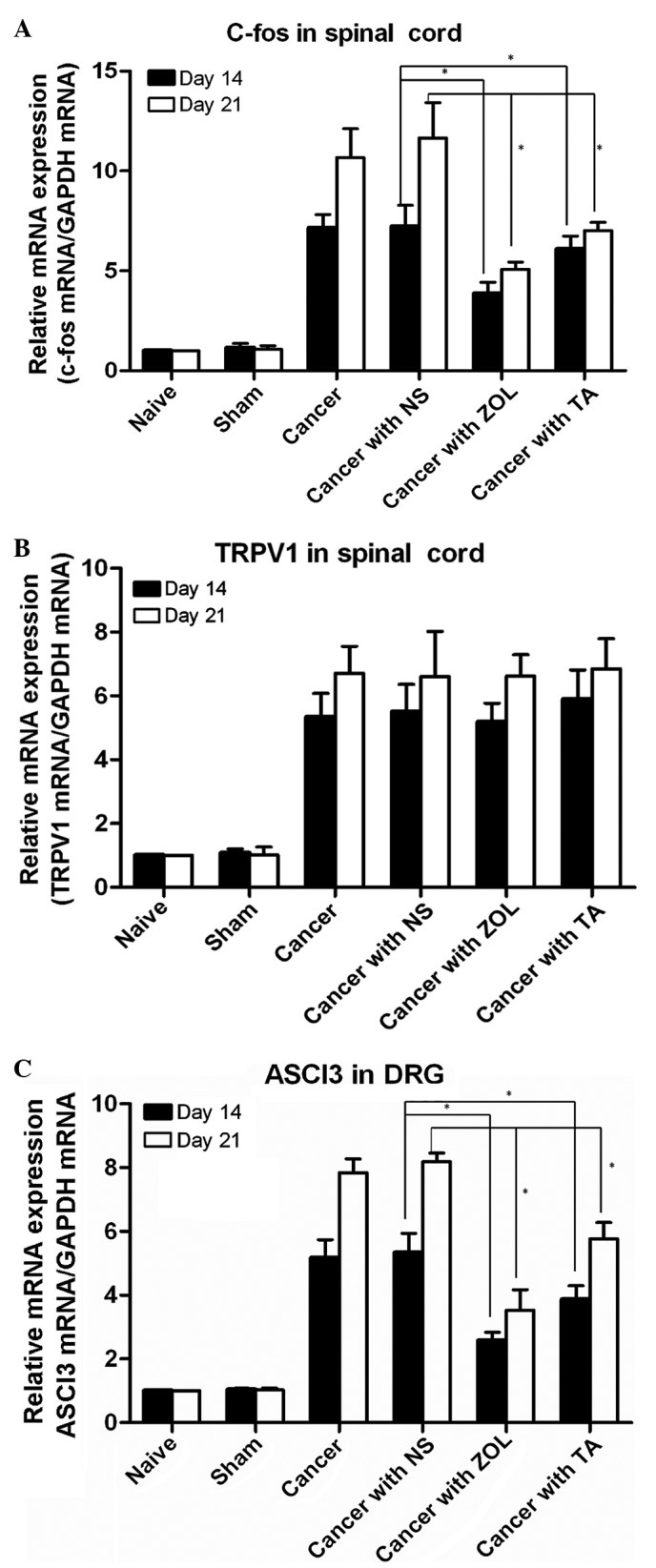
Effects of zoledronic acid and paclitaxel on the relative mRNA expression levels of (A) c-fos and (B) TRPV1 in the spinal cord and (C) ASIC3 in the DRG. ^*^P<0.05. DRG, dorsal root ganglion; NS, normal saline; ZOL, zoledronic acid; TA, paclitaxel.

In order to evaluate the effects of zoledronic acid and paclitaxel on spinal nerve activity, the mRNA levels of c-fos and TRPV1 in the ipsilateral spinal cord, and ASIC3 in the ipsilateral DRG were assessed on days 14 and 21 post-tumor cell inoculation. As shown in Fig. 5, the relative mRNA levels of c-fos and TRPV1 in the ipsilateral spinal cord and of ASIC3 in the ipsilateral DRG in the cancer, cancer with NS, cancer with ZOL and cancer with TA groups were significantly increased, compared with the naive and sham groups (P<0.05). Compared with the cancer group and cancer with NS group, the mRNA levels of c-fos in the spinal cord and of ASIC3 in the DRG in the cancer with ZOL group were significantly reduced on days 14 and 21 post-inoculation (P<0.05). This reduction in the expression of c-fos was observed in the cancer with TA group on day 21 post-inoculation. The mRNA expression of TRPV1 in the spinal cord was not significantly different among the cancer groups.

### Zoledronic acid and paclitaxel significantly reduce the protein expression of c-fos in the spinal cord

**Figure 6:**
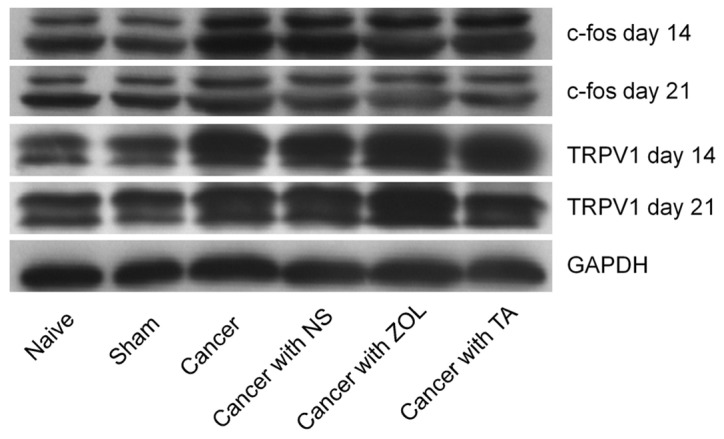
Effects of zoledronic acid and paclitaxel on the protein expression levels of c-fos and TRPV1 in the spinal cord. NS, normal saline; ZOL, zoledronic acid; TA, paclitaxel.

Western blot analysis (Fig. 6) was performed using tissues of rat ipsilateral spinal cord on days 14 and 21 post-tumor cell inoculation, which expressed c-fos and TRPV1 mRNA, as described above. The protein expression of GAPDH was used as an internal control. The results demonstrated that the protein expression levels of c-fos and TRPV1 in the spinal cord were significantly upregulated following tumor cell inoculation, compared with the naive and sham groups (P<0.05). The increase in the protein expression of c-fos was attenuated following treatment with zoledronic acid and paclitaxel on day 11 post-administration (day 21 post-tumor cell inoculation). The protein expression of TRPV1 in spinal cord did not differ amongst the cancer groups.

## Discussion

The chemotherapy-induced peripheral neuropathic pain of paclitaxel has been extensively discussed (11–13). The results of the present study indicated for the first time, to the best of our knowledge, that paclitaxel alleviated the pain induced by bone cancer in a rat model. The results may be due to the fact that the paclitaxel dose (2 mg/kg, once on day 14) was lower than those previously used in chemotherapy-induced peripheral neuropathy investigations (2 mg/kg, every day or every other day for 4–5 injections in total) (11,14–16). Previous studies have demonstrated that the incidence and severity of chemotherapy-induced peripheral neuropathy of the majority of chemotherapeutic agents, including paclitaxel, are dependent on the cumulative dose of the drug (17,18). In the present study, the anti-nociceptive effects in the paclitaxel group were weaker and of later-onset than those in the zoledronic acid group.

NTx is a type I collagen, which is the only collagen present in bone tissue and accounts for 90% of the bone matrix (19). Previous studies have demonstrated that the NTx originated separately from type I collagen and formed a new epitope in the stage of bone resorption, exhibiting high specificity (19,20). NTx is a direct product of osteoclasts (21). In the present study, the anti-nociceptive responses of zoledronic acid and paclitaxel were positively correlated with urinary levels of NTx in the rats. In order to analyze the possible mechanism of this correlation, H&E and TRAP staining of the femoral sections were performed in each group. The extent of infiltration of the bone marrow space by the tumor in the cancer with ZOL group and cancer with TA group did not differ significantly from the cancer control group in the H&E-stained sections. However, in the cancer with ZOL group, more residual trabecular structural elements were observed than in the cancer group. In addition, TRAP staining revealed that the number of osteoclasts present in the cancer with ZOL group was significantly reduced compared with the cancer, cancer with NS and cancer with TA groups. In the cancer with TA group, the number of osteoclasts was similar to that in the cancer group. The results of the present study suggested that alterations in the urinary levels of NTx and the bone pain responses may have been due to the activity of the osteoclasts in the cancer with ZOL group. However, the causes of the changes in the urinary levels of NTx and the bone pain responses in the cancer with TA group cannot be determined from these data. A possible explanation may be a secondary anti-tumor response, although the anti-tumor effects did not result in differences in tumor spaces between the cancer with TA group and the cancer control group.

The present study also analyzed the mRNA expression levels of c-fos and TRPV1 in the spinal cord and ASIC3 in the DRG. The c-fos protein is the product of the immediate-early gene, which is rapidly expressed in neurons following various types of noxious stimuli (22). In addition, upregulation of the c-fos protein has been previously used as a marker for the activation of nociceptive pathways in the spinal cord following peripheral nerve injury (23). In the present study, it was demonstrated that the spinal mRNA expression of c-fos in the ZOL group was reduced more rapidly (4 days post-treatment), compared with that of the cancer with TA group (11 days post-treatment). This suggested that zoledronic acids rapidly relieved peripheral nociceptive injury in bone metastasis, which may have been a consequence of the direct interaction between zoledronic acid and osteoclasts. In addition, the effects of paclitaxel on the remission of noxious stimuli were of later onset, which may have resulted from a secondary anti-tumor response.

ASIC3 is activated by reductions in pH and is important in musculoskeletal pain (24). The present study demonstrated that the spinal mRNA expression of ASIC3 in the ZOL group was reduced more rapidly (4 days post-treatment), compared with the control, suggesting that zoledronic acid may also alter the local acid environment of bone, which can reduce the activation of neurons and further relieve pain.

The TRPV1 channel is voltage sensitive and is activated by acidic pH, the exogenous irritant, capsaicin, and endogenous molecules (25). TRPV1 is also sensitized by a wide range of pro-inflammatory agents, including bradykinin, nerve growth factor, adenosine triphosphate and the chemokines (26). There possibility of cross-talk between the various activators of TRPV1 requires consideration. The results of the present study revealed no significant differences in the mRNA and protein TRPV1 expression levels in the ZOL and TA groups, compared with the control group. This suggested that the treatment outcome of zoledronic acid or paclitaxel on bone cancer pain is not the result of osteoclasts, an acid environment or tumor progression alone, and that various factors are involved in bone cancer pain, which may interact and offset each other.

In conclusion, the present study demonstrated that a low dose paclitaxel was observed led to a weaker and delayed anti-nociceptive effect on bone cancer pain, compared with zoledronic acid. Zoledronic acid may directly interact with osteoclasts and alter the local acidic environment of bone, which results in rapid relief from peripheral nociceptive injury in bone metastasis. In addition, urinary levels of NTx may predict the anti-nociceptive responses of zoledronic acid and paclitaxel in rat models of bone metastases.
